# Migration Medicine: Notes on a Young Science^†^

**DOI:** 10.4269/ajtmh.18-0587

**Published:** 2018-09-04

**Authors:** Patricia Frye Walker

**Affiliations:** HealthPartners Center for International Health, St. Paul, Minnesota

Thank you very much, Marty [Cetron], for that very generous introduction. I want to thank you most of all for your many years of friendship, and your visionary leadership in the field of global migration.

I also want to begin by thanking my mentees, many of whom are in the audience today. The future of migration medicine is in your hands. It has been such a privilege to have an influence on your careers and to watch your incredible impact on tropical medicine and refugee and immigrant health worldwide. I want to thank my family for their lifelong encouragement and most of all, my wife, Becky Enos, for 25 years of unflagging support and love.

“Migration Medicine: Notes on a Young Science.” The title is a takeoff from one of my favorite books as a young resident physician: Lewis Thomas, *The Youngest Science: Notes of a Medicine Watcher*.^1^ The title captures for me what I wanted captured. A sense of humility about what we do not know about migration medicine, as well as a sense of curiosity and hope for the future of the field. Today, I will paint a picture of migration medicine as a young science, focusing on the group of migrants who are refugees and offer you my perspectives as a clinician and medical educator in the field.

But before I begin I want to take this opportunity to talk a little bit about the Society this year and what we’ve been doing. It’s been a truly remarkable time and I’ve been very proud of the work that we’ve collectively done on behalf of global health and tropical medicine in the past year.

What were my hopes for the presidential year? I had a few plans, and tried to hit the ground running. I worked to continue to advance our strategic plan as determined by the Council and recent presidents, including Dr. Alan Magill, one of my mentors, Dr. Chris Plowe, and Dr. Steve Higgs. We have done some amazing work. We had a Trainee Task Force led by Dr. David Fidock, Dr. Stephanie Yanow, and Dr. Julian Rayner. The outcome of which, I am happy to say, is that we will have two trainees as full voting members of the Council in the future.

Our International Task Force was led by Dr. Abdoulaye Djimde and Dr. Nicole Achee. We will be supporting a Digital Education Committee and also continue our support for regional meetings. Dr. Djimde is going to be leading a meeting in Mali, for example, in the next few months.

Our Awards Task Force completed their work. As you know we have two new awards this year: the first Alan Magill Award Fellowship recipient is Pedro Aide and our new Society-level medal named after a woman pioneer in the field, Clara Ludlow.^2^ I want to thank Dr. Steve Higgs for his visionary leadership in regards to the Ludlow Medal. This required a lot of work on the part of many members of the Society, members of the Council and, of course, of the staff.

We also have a new Chair of Clinical Tropical and Travel Medicine Education Programs, Dr. John Sanders. We are taking a fresh look at our CTropMed^®^ examination and courses, our Clinical Tropical Medicine Update course, and are talking again about pursuing a subspecialty–board certification in clinical tropical medicine. This is something Dr. Michele Barry, Dr. David Hill, and others were working on years ago.

We also changed the way that the Council works. I’m trying to have more conversation, leveraging the expertise of the Council; I must say it has given everyone a chance to have more of a voice, which is a good thing.

As Dr. Cetron mentioned, we strengthened our migration medicine focus with the “Refugee Journey to Wellbeing”^3^ exhibit at last year’s annual meeting. We held a Tropical Medicine Update Course in concert with the North American Refugee Health Conference that was sold out. This connected primary care providers who are seeing patients with clinical tropical medicine problems, to members of American Society of Tropical Medicine and Hygiene (ASTMH) who are experts in clinical tropical medicine.

Our Migration Medicine Pre-Meeting Course this year was organized by two Society members, Dr. Christina Greenaway and Dr. Susan Kuhn, and in 2018 we are co-sponsoring a Migration Medicine conference with the International Society for Travel Medicine in Rome, Italy.

One week before our Annual Meeting last year, the U.S. Presidential election was held. The election of Donald Trump almost immediately had the potential for tremendous impact on the future of global health funding including threats to the NIH, CDC, USAID, and, as you know, the proposal to close the Fogarty International Center (FIC). The travel ban had immediate impacts on science diplomacy, threats to sanctuary cities and Deferred Action for Childhood Arrivals, as well as the reduction in the refugee admissions ceiling to 45,000. As a result, this year’s activities definitely included other duties as necessitated by circumstances. I was very glad that, as Dr. Cetron mentioned, my father taught me to “always have an alternate runway and enough gas to get there.”

Of course, the good news is that ASTMH has advocacy as one of the pillars of our strategic plan. I want to just highlight a few of the things we’ve been doing the last 6 months in terms of advocacy:

Almost presciently, after the election and before the first travel ban, we published an editorial on the importance of evidence-based policies on migration.^4^ This was widely distributed; the key message was the need to maintain established U.S. policy toward human migration and global health which is evidence-based and upholds the values of compassion and international human rights law.

President Trump’s first Executive Order on immigration brought tremendous response from the legal, the business, and the scientific community. Silicon Valley, including Apple, Google, and Uber all responded with statements. This was the first of several interventions by the courts in the ensuing months temporarily halting these Executive Orders. We have certainly seen democracy in action this year, and civil society speaking out.

I woke up the morning after the first travel ban and felt I had to write something in response, focusing on what I know about the vetting which refugees go through before U.S. arrival, as well as the privilege of working in refugee health.^5^ I will say more a little bit later about the health vetting of refugees specifically, but I also want to call out, as a clinician, the impact that many of us have seen in our clinical practices with more anxiety, more depression, more post-traumatic stress disorder on the part of both refugee and non-refugee patients as they feel less welcome and more uncertain about their future.

American Society of Tropical Medicine and Hygiene responded to the travel ban outlining its unintended consequences.^6^ This was widely distributed and followed by Society members contacting the White House as well as updating our website homepage. We joined with 170 other leading scientific organizations in signing a letter to Donald Trump stating that we stand ready to help his Administration to craft an immigration and visa policy that advances our prosperity as a nation while staying true to our foundational American principles as a nation of immigrants.^7^

The academic community also responded, outlining the detrimental effects on medical training and healthcare.^8^ *New York Times* journalist Donald J. McNeil, Jr. wrote a story on one of our University of Minnesota internal medicine residents from Syria, who was worried about fellowship matching.^9^

Some statistics from the Education Commission on Foreign Medical Graduates are telling. Twenty-four percent of U.S. doctors are international medical graduates and 21% of applicants to the Match in 2016 were international medical graduates.^8^

When the second travel ban occurred we responded again as a Society, expressing continued concerns on the new Executive Order restricting travel, emphasizing that, as we know, academic medicine by its nature involves bilateral exchange.^10^

And then in March, with the proposed serious cuts to the NIH budget and others, we reacted immediately – can you tell we were busy the first few months of the year? – with continued statements.^11^ I won’t read through them but will comment for Society members that whenever something would happen in Washington, we would convene as an Executive Committee and make decisions as a group in terms of statements which were written. Our policy is to always issue statements which are nonpartisan and evidence-based.

In May we partnered with the Infectious Disease Society of America and Research!America on a webinar on the economic value of the FIC. In September, in an effort led by our editor-in-chief, Phil Rosenthal, the *American Journal of Tropical Medicine and Hygiene* featured four invited editorials in support of the FIC.^12^

As we all know there is power and strength in advocacy. The FIC has been saved with a slight increase in its budget. As of last month we had a $2 billion increase to the NIH, but must maintain vigilance in regards to funding issues. While this success is a result of the efforts of many constituencies, I think that ASTMH is seen more and more as a significant expert voice on funding for global health and tropical medicine. We bring that expert voice to the conversation and we do this not just for the U.S. but for the global community.

I had the honor many years ago to sit on a global health panel with Dr. Bill Foege and have always been so inspired by his words, which still ring true today, that every public health decision involves political decisions.^13^

This work, summing up what we’ve done this year as a Society, was in addition to all of the usual work: all the membership support, the Annual Meeting, the Pre-Meeting Courses, the Update Course, the *Journal*, our media and public relations work. I got a clue as to the ASTMH staff’s level of dedication when I met Karen Goraleski in Chicago for my orientation to the role of President and saw her license plate: “*TROPMED*.” Please join me in a round of applause for the ASTMH team.

Migration Medicine, for the purposes of this talk, I am defining as the field of medicine, clinical care, education, research and public health public policy as it relates to the care of international migrants. Migrants are defined by the United Nations (UN) as someone who has lived outside of their country of birth for more than a year.

Migration has been a constant dynamic in human history. As innate to us as breathing is our desire to explore, to move toward the future and particularly to a better future for us and our children. People have been migrating since the beginning of human history to escape natural disasters, war and dictatorships, and to seek a better life for themselves and their family. These flows have greatly benefitted the world, leading to cultural, societal and intellectual advances. They have built the United States and they are fundamental to our national identity.

Scientists have noted that we may be more aptly called *homo prospectus* because research suggests that looking into the future consciously and unconsciously is a central function of our large brain.^14^

Just a few comments on the numbers of migrants worldwide are instructive. Tourist travelers, 1.24 billion,^15^ far outnumber migrant travelers and have obvious implications for global health security. At 150 million, migrant workers accounted for 65% of all international migrants in 2013.^16^ The implications for global health security in relationship to both migrant workers and travelers are profound. Of course, members of this Society live and breathe this work every day.

Our previous President, Dr. Chris Plowe, who is here tonight, wrote a recent op-ed in *The New York Times* about the relationships of multi-drug-resistant malaria to migrant workers in the Mekong Region.^17^ The importance of intact healthcare systems and providing care for legal or illegal migrant workers cannot be overemphasized. Thailand’s health insurance program for migrant workers is an amazing example of a rational and forward-thinking response to human migration and health issues. Migrant workers also contribute significantly to the GDP of many countries, contributing more than 20% in 10 countries, with the World Bank estimating that $582 billion was generated in 2015 by migrant workers worldwide.^18^

What then has changed about human migration? What has made it such a politically charged subject which has led to a rise in nationalism and xenophobia? It is due, in part, albeit only in part, to the numbers: human migration has more than tripled since 1960, rising from 77 to 244 million,^19^ as well as the speed of travel. The speed of travel impacts us particularly from a global health security perspective: the ability to circumnavigate the globe in a little over 1 day as opposed to by boat over 1 year in previous centuries.

Migration is also much more complex: within and between countries for work, legally, or illegally, including human trafficking. It is also circular, such as the visiting friend and relative traveler, and it involves children. Many of you saw this very sad picture of Alan Kurdi, a 3-year-old boy who was trying to flee the war in Syria hoping to join relatives in Canada. He and his mother and brother all drowned when their small inflatable boat capsized as they left Turkey.

Migration also has a profound impact on health. These slides are courtesy of my tropical medicine colleague, Dr. Alexia Knapp. I’ll share with you a very brief clinical case:

A 33-year-old man presented with signs and symptoms of cutaneous leishmaniasis. His tissue block was sent to CDC, confirming that he had *Viannia* subgenus. They were not able to confirm the species, but Dr. Knapp knew that he was from Somalia, and that there is no cutaneous leishmaniasis in Somalia. So she asked, “Tell me your story.”

He was from Mogadishu and worked as a street vendor prior to leaving. He fled because of fear of persecution. His father and brother had been recently murdered. His story was absolutely incredible. He described to Dr. Knapp a known pipeline to the west, paying a smuggler $15,000 for safe passage to the U.S. He spent 3 months flying from Nairobi to Sao Paulo, Brazil, and then 3 months going through South and Central America. He described going through the Darien Gap, the missing link in the Panama Highway, 100–160 km of undeveloped swamp land and forest within Panama’s Darien Province and northern Columbia. It is a place where no road connects North and South America. His highest risk for exposure to leishmaniasis on this journey was probably in Panama, probably in the Darien Gap. It also could have been at the Costa Rican-Nicaraguan border.

And just when Dr. Knapp made this patient’s diagnosis, the first travel ban occurred in the U.S. this past January. He disappeared; we don’t know where he is, and think he may have gone to Canada. And as you know, hundreds of African and other migrants are fleeing across the U.S. to Canada, some dying or suffering severe frostbite along the way.^20^

His is a poignant example among many that we must do a better job of making migration safe. At a UN summit in September of 2016, the world came together around a plan called the New York Declaration on Refugees and Migrants. I commend that report to you, which has some outstanding recommendations.^21^ Among its many recommendations was the recommendation that we must develop a global compact for safe, orderly, and regular migration.

In terms of forced migration: the numbers are really staggering. Sixty-five million forcibly displaced, 21 million refugees, more than half of those less than 18. Thirty-four thousand people are forcibly displaced every day.^22^ Since the civil war began in Syria, approximately 7 million have been displaced, 5 million fleeing to nearby countries, and 1 million requesting asylum in Europe,^23^ overwhelming healthcare systems. And of course, most recently in the last few months, ethnic Rohingya have been fleeing Myanmar. The UN has called this a textbook example of ethnic cleansing.^24^ I tell trainees, “The sad reality is that you will have guaranteed job security if you’re interested in a career in refugee health.”

When I began working in refugee health in 1979, the modern refugee protection movement was 58 years old, if one dates it to 1921. Certainly our response as human beings has been to protect each other since antiquity. However, formal, international, legal protection for refugees just began with the League of Nations in 1921.^25^ The convention on the international status of refugees in 1933 was the first time this principle of non-refoulement – not to push a person out of a country if it is not safe for them–acquired the status of international treaty law.^26^

In 1938, the U.S. President, Franklin Delano Roosevelt, criticized for not doing enough about the Holocaust, called for a meeting in Evian, France, outside of the context of the League of Nations. The good news was this was the first time that the concept of protecting people while they are in their home country, such as German Jews, was accepted. The bad news was that 33 countries participated in the Evian Conference, and only Costa Rica and the Dominican Republic agreed to increase their quota for Jewish immigration. The conference thus proved to be a useful propaganda boost for the Nazis,^27^ and it’s a story you should remember, as I will connect to it again a little bit later.

After World War II, the International Refugee Organization (IRO) was formed, established by the U.N. General Assembly to help resettle the massive refugee movements created by World War II. The IRO thought they would complete their work within 5 years. When they realized this was not feasible, the U.N. commissioned a Study of Statelessness,^28^ which remains a key document in the modern history of refugee protection. It looked at issues such as, “Can I safely travel through a country? Can I work? Can my kids get educated? Do I have to work in the military in that country and would I be taxed?” This study served as the main elements of the U.N. Convention Relating to the Status of Refugees in 1951.^29^

As you know, a refugee is someone who, owing to a well-founded fear of persecution, cannot safely go back home.^30^ They are very different from other migrants, particularly economic migrants who choose to move. Refugees have to move if they are to save their lives or preserve their freedom. They are particularly vulnerable. They often have complex healthcare needs and many have been through unspeakable trauma.

The U.S. has a long history with migrants. We are a nation formed by people fleeing persecution, particularly religious persecution, and then in the last 70 years, of course, accepting displaced Europeans, creating laws assisting those fleeing communism, from Cuba, and then the U.S. Refugee Act of 1980,^31^ in and of itself an interesting story.

How did it come about? It was, in part, advocacy and appealing to our shared humanity. In 1979 the U.S. spearheaded an international conference at Lake Geneva near Evian in France, on the plight of hundreds of thousands of refugees fleeing the communist victory in Southeast Asia. In an emotional keynote speech, Vice President Walter Mondale compared the gathering to the Evian conference of 1938, which he said failed the test of civilization. Mondale pleaded with the delegates to join the U.S. in rescuing what he called “the Asian boat people.” He said, “History will not forgive us if we fail; history will not forget us if we succeed.” The speech was widely credited with inspiring many countries to take part in the rescue of Vietnamese refugees.

The United States stepped up to the crisis. Mondale’s chief speechwriter, Martin Kaplan later recalled, “It was one of those rare occasions where words may actually have saved lives.”^32,33^ That is a brief background about the refugee resettlement movement in the U.S. and internationally.

The United States is a nation of immigrants: 99.1% of us are immigrants or their descendants. Numbers can help provide perspective: 60 million Americans travel abroad every year, and 60 million enter the U.S. Most of those are tourists, business travelers or short term visitors. About one million immigrants become legal permanent residents or green card holders every year, and about 70,000–90,000 are accepted as refugees. Since 1975 the U.S. has accepted about 3.25 million refugees. We peaked in 1980, right after the Vietnam War, at over 200,000.^34^

In medicine we think about evidence-based clinical guidelines and I’d like to speak briefly to the importance of evidence-based messages regarding refugees in four key areas:

The first is a historical perspective about the numbers of displaced. The truth is that this is not a new problem. The number of displaced persons worldwide has been almost this high in the past. Forty million people were displaced after World War II, and with the partition of India and Pakistan, 14 million were displaced in 1947.^35^

The number of refugees has also been this high. In 2015 there were 21 million refugees in the world. In 1992 there were 20.6 million, when the global population was two-thirds of its current number.^36^ It puts the request to the developed world from the U.N. to accept 1.2 million refugees in perspective. Less than 1% of the world’s refugees are accepted for resettlement annually. That 1% can be particularly traumatized but also particularly resilient. I vividly recall returning from the Thai-Cambodian border in 1979 and hearing people discuss those “poor illiterate refugees,” while at the time I was meeting the prima ballerina of the National Cambodian Dance Studio, and the leading sculptor of the nation who said, “I’d like to sculpt something to thank America.” And, one of the few physicians who survived the killing fields, my friend Dr. Haing Ngor, who went on to win an Oscar for his portrayal of Dith Pran in the movie *The Killing Fields*.

The U.S. has approximately one million legal migrants every year, very similar to the one million who came in 1910. The U.S. had more first-generation immigrants from 1860 to 1920 than now.^36^ This is a really important issue, as media portrayal of “overwhelming numbers” of migrants can impact either positively or negatively on receiving countries, their citizens and politicians.

In a 2017 article in *Nature* about human migration, the social scientist, Nando Sigona commented, “The alleged increase in migration and forced displacement tells us more about the moral panic on migration than the reality.”^36^

Another important evidence-based message is about the economic impact of migrants. As it was preparing for an October 1 deadline to determine the number of refugees to come to the U.S., the Trump Administration rejected the findings of a study by the U.S. Department of Health and Human Services which found that refugees brought in $63 billion more in government revenues over the past decade than they cost.^37^ And of course the easiest myth to address quickly, the odds of a fatal terror attack in the United States by a refugee are estimated at 3.6 billion-to-one.^38^

Refugees are the most heavily vetted of any people who enter the U.S. They face an 18- to 24-month processing period. I want to speak specifically for this Society to the infectious disease risk. Remember that migrants only represent a very small fraction of international travel to the U.S.; as an example, 95% of Zika cases are travel related.^39^ I want to briefly discuss the example of the work of the CDC Division of Global Migration and Quarantine led by Dr. Martin Cetron, as an example of a best practice in regard to concerns about planned migration and risk of infectious diseases.

The CDC has been addressing healthcare needs of refugees for many years.^40^ They have responded to multiple outbreaks among U.S. bound refugees of measles, rubella, varicella, cholera, hepatitis A, N’yong-n’yong fever and multi-drug-resistant tuberculosis (MDR TB) with importation of infectious disease to the U.S. and secondary domestic transmission including MDR TB and congenital rubella. Such outbreaks tax local health departments, halt resettlement and cost hundreds of thousands of dollars. But over the past two decades, I’ve been watching this and have just been so amazed by CDC’s work, a strategy of surveillance, improving overseas diagnostic capacity, and pre-departure treatment that has resulted in improving patients’ lives and reducing imported diseases. This work, honestly, is worthy of an entire lecture. Suffice it to say that refugees are the most heavily vetted immigrants from a health perspective, and I think they can be a role model for better screening of international migrants in general.

One example is the implementation of the new CDC technical instructions for screening of U.S. bound migrants, which involves enhanced screening with chest X-rays, sputum AFB, and culture. It has resulted in increased case detection abroad and fewer diagnoses of TB in the foreign born within the first year after arrival.^41^

Another example is intestinal parasites. Since CDC implemented presumptive pre-departure albendazole treatment of refugees, the domestic diagnosis of parasites has dropped dramatically.^42^ The estimated potential cost savings is $92 million.^42^ I used to order multiple stool specimens looking for *Strongyloides* in refugees with eosinophilia, for example, before we were able to obtain serology. There are similar data that exist for presumptive malaria treatment^43^ and an expanded vaccine preventable disease program,^44^ which the CDC, the State Department, and the International Organization for Migration have implemented for U.S.-bound refugees. This work changes practice, and I joke with Dr. Cetron that the CDC Division of Global Migration and Quarantine has made my life in clinical tropical medicine much less interesting.

Not that we have all the answers. We are currently experiencing an outbreak of MDR TB in Minnesota. We had zero to three cases per year between 2011 and 2015, nine cases last year, and eight cases thus far this year, with the Hmong community particularly affected.^45^ Migrants will continue to bring in infectious diseases and we must be prepared domestically. Certainly globalization does have its challenges, including the need for prepared domestic providers and healthcare delivery systems.

We also had a measles outbreak in Minnesota last year impacting primarily the Somali community. This outbreak, as you know, was about unvaccinated children, not about specific communities or ethnic groups. We know that the outbreak was related to plummeting vaccination rates after Somali children were diagnosed with autism and the discredited physician Andrew Wakefield visited the state. With a great deal of community outreach, vaccination rates are up significantly and the outbreak is over.^46^

A few final comments on what have we been doing in refugee and migrant health. I’ve mentioned our upstream public health interventions, some exciting work on developing and updating guidelines, and tremendously improved communication between the international community and those of us on the domestic side receiving refugees. Fifteen countries, for example, now have guidelines. We have an electronic disease notification system that connects international and domestic medical records. Whenever there is an outbreak occurring with US bound refugees internationally, we hear about it on the domestic side. We’ve been educating providers–many ASTMH members in this room have been involved with that, and conducting research on refugee health. Certainly more is needed, particularly in Europe now, for example. I was pleased to hear this week from a Fogarty colleague that they are holding a meeting in the next few months on research issues during humanitarian crises.

As Dr. Cetron mentioned, we’ve launched national CDC Refugee Centers of Excellence which have been very exciting. For example, one of the new guidelines we are developing concerns screening refugees for cancers related to infectious disease which are more common in migrants, such as hepatitis B and hepatoma, *Helicobacter pylori* and gastric cancer, human papilloma virus and cervical cancer, or biliary flukes and cholangiocarcinoma. CDC is working on the concept of a reverse yellow book. We all know the CDC Yellow Book, advising clinicians on pre-travel care for individuals traveling internationally. The reverse yellow book will be a new interactive web-based guide that tells clinicians in the United States that if a patient is from the Congo, for example, what infectious disease screening they should consider for that patient. That is really exciting new work and I think it is more than time for us to do a better job leveraging information systems and making them more user-friendly for clinicians who are seeing migrants worldwide.

Refugee healthcare can be a model for best practices in migration medicine which are safe, orderly, planned, with upstream interventions and coordinated care, evidence based, and getting better in that regard, and with prepared providers and healthcare systems.

I’d like to wrap up by imagining our future in migration medicine. I’d like to imagine a world where we work for peaceful resolution of international conflicts so that those of us in the field don’t have jobs. I’d like to imagine a world where we honor key principles of international refugee law, where governments, international organizations and non-governmental organizations are prepared for high volume, long-term tragedies such as the Syrian conflict, where upstream public health interventions are supported with adequate funding. I imagine a world where we have universal health care access. (I was very pleased to read recently that World Health Organization Director General Dr. Tedros’ number one goal is universal health care access.) A world which views refugee situations as the indescribable human tragedies which they are and which responds with generosity and compassion. As Pope Francis said, “refugees are not the danger, they are in danger.”^47^

I’d like to imagine a world where leading governments respond by increasing refugee acceptance numbers. When Canadians saw the picture of Alan Kurdi lifeless on the beach, they responded by accepting 25,000 Syrians in 4 months.^48^ I imagine a world where transitions of care are seamless as refugees move from country of first asylum to resettlement communities. I imagine a world where providers are trained in the body of knowledge which encompasses refugee and immigrant healthcare. American Society of Tropical Medicine and Hygiene can and does play a significant role in regards to this goal.

I am very pleased that there is a lot of knowledge that did not exist 35 years ago when I began in the field, and also that people like Dr. Alexia Knapp will say, “Where were you born and where have you traveled?” and they will know what to do with the answer. And where anything less than that is unacceptable from a health equity perspective.

I imagine a world where patient outcomes are better because we know the differential diagnosis of diseases seen in the tropics. A world where we remember that unlike migration 200 years ago, when people left and never went back home, that migration is now circular. And that we routinely ask, “Are you planning to go back home?”

I hope for a world where scientists are not afraid to be advocates. We are, after all, key witnesses to health disparities and suffering in the world from tropical diseases. I was at a conference with Dr. Cetron at Dartmouth this April and was inspired by Lancet editor Richard Horton’s words when he spoke about the journal *The Lancet*, saying, “I realized the academic community was this massive untapped resource. Advocacy has been a focus for us as a journal since our series on child survival in 2000 and we’ve never looked back.”

And of course I am inspired by the words of Einstein many years ago that “Nationalism is an infantile disease. It is the measles of mankind.”^49^

I also believe that if we stay true to our core values in global health, that should help guide how we make decisions about resource allocation and global health research, teaching, and clinical care which focuses on equity and compassion. Most recently, the Trump Administration lowered the refugee quota to 45,000, the lowest number since the Refugee Act of 1980.

What’s in a number? I think what’s in a number is a challenge to our identity as a nation, and also I believe it’s a denial of our core values as human beings. And so I must ask, who will speak out like Walter Mondale did at the Lake Geneva conference in 1979, which prompted the Refugee Act of 1980? How many more children like Alan Kurdi must die before we act? I believe the U.S. should set the U.S. refugee ceiling at 110,000 or higher every year. Humanity will benefit and the U.S. will be better as a nation as a result.

I feel that if one believes one can influence one’s future, then optimism is a moral imperative. And the future is in great hands. This photograph is of some of my mentees doing amazing work around the world. I could tell you a lot about each one of them, but you can ask them as well. Many of them are in the audience.

Just last month, my wife and I were in New York City celebrating our 25th anniversary and we went to the Museum of Modern Art. Throughout the museum was spectacular artwork by immigrants to America with an inscription under many of the pieces such as this one which said, “This work is by an artist from a nation whose citizens would be denied entry into the United States according to recent presidential executive orders.” I think our January AJTMH editorial was correct in saying that perhaps the Statue of Liberty inscription is outdated and it should just be changed to, “Give me your energetic, your geniuses.”^50^ As Frank Bruni said in an op-ed piece last month in reference to immigrants, “They come with the sort of hunger and a kind of gaze that doesn’t subtract from what those of us already here have but instead add to it. They give us insights, invention, art. Embracing their genius is the genius of America.”^51^

My final thought about our Society and the important role that we can play in this regard, is that migration medicine is a young science that needs concentrated international focus. In preparing for this address, I realized that international human rights law is probably ahead of migration medicine in terms of best practices, but that both need a lot of attention. We must answer fear and ignorance with scientific evidence and our core values.^52^ Humanity and global health security are counting on our responses.

Thank you very much.

**Figure:**
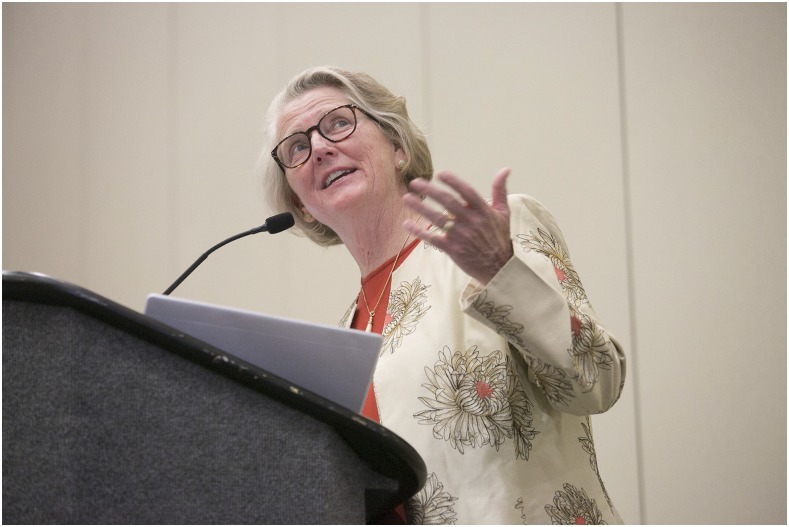
**President Patricia F. Walker, MD, DTM&H, FASTMH, delivers her President’s Address during the 2017 Annual Meeting in Baltimore, MD.**

**Figure:**
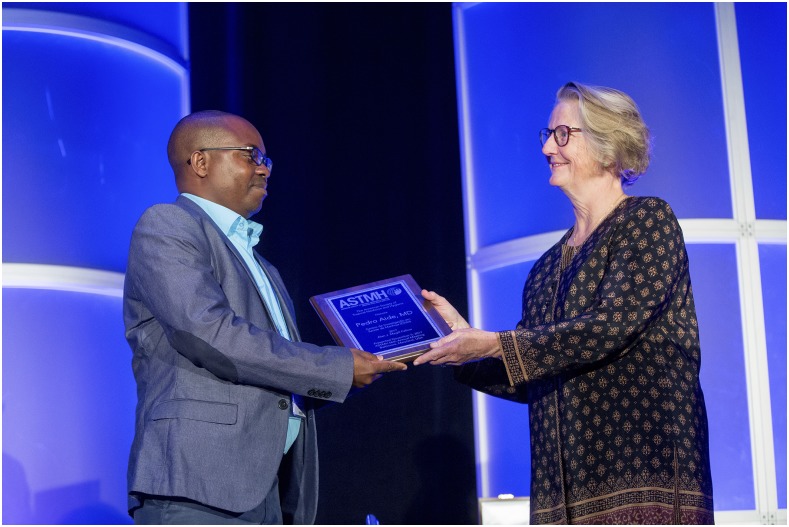
**President Patricia F. Walker, MD, DTM&H, FASTMH, presents a commemorative plaque to Pedro Aide, MD, MSc, PhD, of Mozambique, the first recipient of the Alan J. Magill Fellowship, during the opening ceremony at the 2017 Annual Meeting.**

**Figure:**
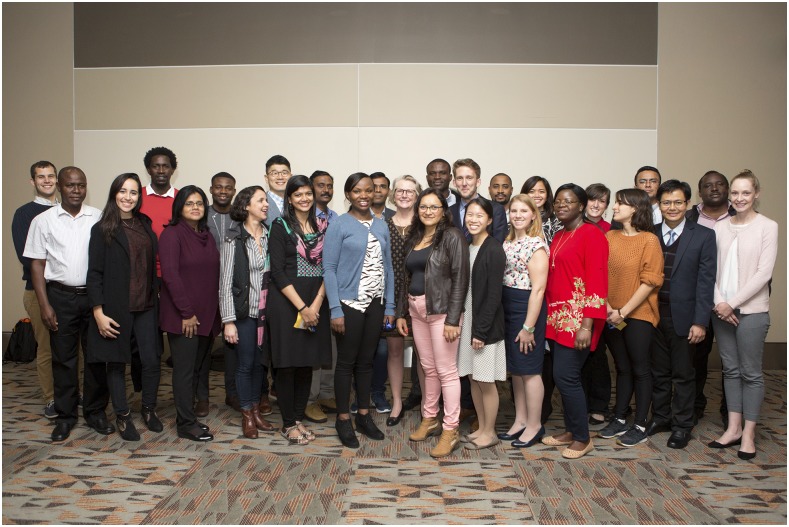
**President Patricia F. Walker, MD, DTM&H, FASTMH, (center) poses with the 2017 American Society of Tropical Medicine and Hygiene Travel Award Recipients at the Annual Meeting.**
